# *In vitro* Propagation of Arbuscular Mycorrhizal Fungi May Drive Fungal Evolution

**DOI:** 10.3389/fmicb.2019.02420

**Published:** 2019-10-22

**Authors:** Vasilis Kokkoris, Miranda Hart

**Affiliations:** Department of Biology, University of British Columbia, Kelowna, BC, Canada

**Keywords:** arbuscular mycorrhizal fungi, fungal domestication, fungal evolution, *in vitro* propagation, transformed root cultures

## Abstract

Transformed root cultures (TRC) are used to mass produce arbuscular mycorrhizal (AM) fungal propagules *in vitro*. These propagules are then used in research, agriculture, and ecological restoration. There are many examples from other microbial systems that long-term *in vitro* propagation leads to domesticated strains that differ genetically and functionally. Here, we discuss potential consequences of in TRC propagation on AM fungal traits, and how this may affect their functionality. We examine weather domestication of AM fungi has already happened and finally, we explore whether it is possible to overcome TRC-induced domestication.

## Introduction

Domestication of plants and animals has been a hallmark of the Anthropocene (Zeder, 2006), resulting in altered morphology, decreased genetic diversity, altered behavior, and altered function in the domesticant. Such far reaching changes are necessary to maintain the domesticated state but can present a risk to food security (Chen et al., 2015; Whitehead et al., 2017; Egan et al., 2018), and in some cases, to the status of the domesticated species (Pearce, 2003; Ploetz, 2003). For example, domestication of wild bananas led to a sterile and genetically homogeneous cultivar that now faces extinction (Pearce, 2003; Ploetz, 2003). Similarly, decreases in genetic diversity as a result of domestication has been well documented in many crops, including common bean (Bitocchi et al., 2013), rice (Ram et al., 2007), wheat (Reif et al., 2005), soybean (Hyten et al., 2006), and pear (Nishio et al., 2016). This decrease in genetic diversity is often associated with losses of functional traits such as herbivore resistance (Chaudhary and Bhupendra, 2013), reduced immune function (e.g., Honey bees, López-Uribe et al., 2017) or even behavioral changes such as loss of immigration ability in monarch butterflies (Tenger-Trolander et al., 2019).

The effect of domestication on ecological competence is not novel in mycology (Jinks, 1952; Roper et al., 2011); plant pathogens lose pathogenicity when kept in culture for extended periods (Naiki and Cook, 1983), and domestication of *Saccharomyces cerevisiae* created yeasts without the ability to reproduce sexually or survive outside of laboratory conditions (Gallone et al., 2016). *Aspergillus oryzae* diverged from a pathogen to become a commercially important fermenter and subsequently lost genes related to pathogenicity (Machida et al., 2005). Such losses may result from bottleneck effects and environmental selection, especially if the system used in cultivation does not represent the conditions under which they originally evolved (see review by Douglas and Klaenhammer, 2010).

**Figure 1:**
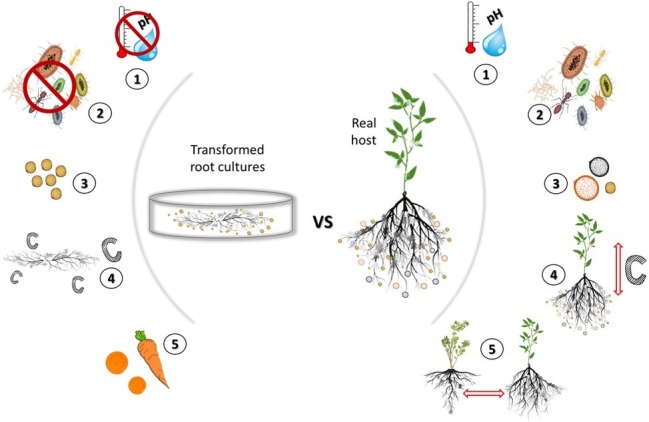
Comparison of the artificial growing environment of transformed root cultures (TRC) to a natural environment. While TRC can produce uncontaminated and abundant AM fungal propagules, they consists a highly artificial and eutrophic environment that lacks environmental stimuli. **(1) Lack of abiotic stimuli**: Petri dishes are kept sealed to retain moisture and are propagated in the dark at a stable temperature that benefits the AM fungal productivity (propagule production). In a natural environment, there is continuous fluctuation of temperature, and moisture as well as high spatial variation in pH but also in multiple other abiotic factors. Such abiotic factors can affect the AM fungal growth as well as the mycorrhizal response (MR) (Wang et al., 1993; Heinemeyer and Fitter, 2004) and have seen to alter the gene expression of fungi (Meyer et al., 2017). **(2, 3) Lack of biotic stimuli**: the main benefit of TRC is the ability to grow AM fungi under sterile conditions that allows for uncontaminated propagation of the desired species/isolate. Each TRC contains only a single isolate of AM fungi. AM fungi have antagonistic and synergetic relations with other soil microbes (Mar Vázquez et al., 2000) as well as with other AM fungi (Wilson, 1984; Engelmoer et al., 2014). The TRC environment lacks biotic interactions that when present can lead to the production of multiple chemical compounds (Naiki and Cook, 1983; Gallone et al., 2016) that confer stress resistance or can enhance the metabolic activity of the engaged microbes (Ola et al., 2013). **(4) Unrestricted carbon flow**: TRC lack a shoot and therefore carbon flow to the AM fungus is consistent and continuous (Fortin et al., 2002) in contrast to a real plant where carbon flow fluctuates daily and seasonally between shoot and root (Lippu, 1998). **(5) Lack of host diversity**: in nature, AM fungi can interact simultaneously with multiple host. Host identity can alter significantly the gene expression of AM fungi (Mateus et al., 2019) and alter the progression of the symbiosis (Angelard et al., 2010). The dramatic reduction of host diversity and continuous re-cultivation using a sole host [most commonly carrot (*Daucus carota*)] may affect the function and genetic diversity of the cultured AM fungi.

Arbuscular mycorrhizal (AM) fungi are obligate biotrophs that participate in an ancient symbiosis with plants (Smith and Read, 2008; Brundrett and Brundrett, 2009). Through this symbiosis, AM fungi provide plants increased access to soil resources in return for carbon in the form of sugar and lipids (Luginbuehl et al., 2017). Besides the nutritional benefit to the plants, AM fungi can also increase plant tolerance to environmental stress [e.g., water (Ruiz-Lozano and Aroca, 2010), salinity (Porcel et al., 2012), and heavy metals (Díaz et al., 1996)]. AM fungi are known to stimulate plant photosynthetic activity (Boldt et al., 2011) and enhance plants’ disease resistance (Pozo and Azcón-Aguilar, 2007; Jung et al., 2012). Because of these benefits, considerable effort has focused on finding ways to propagate and study these fungi for potential applications including agriculture, landscaping, and landscape restoration (Sawers et al., 2008; Berruti et al., 2016). One of the most successful methods of propagating clean material employs the use of transformed root cultures (TRC) (see sidebar#1 for information on TRC) (Mosse and Hepper, 1975; Bécard and Fortin, 1988; Stockinger et al., 2009; Rosikiewicz et al., 2017). While this method is efficient for producing uncontaminated propagules, it represents a highly artificial environment and could potentially lead to domesticated AM fungal strains (Figure 1).

Sidebar#1 **Transformed root cultures.**

TRC involves infection of a “hairy root” culture with an AM fungal spore. A hairy root culture is the product of gene transfer from the root-inducing (Ri) plasmid of the parasite *Agrobacterium rhizogenes* into the genome of a host plant (Willmitzer et al., 1982). The Ri plasmid contains genes that increase rates of both cell division through increased cytokinins production and cell elongation through auxin production. The transformed root also produces opines used as a food source by the colonizing bacteria. After eliminating the bacterium with antibiotics, the resulting plant is a proliferation of “hairy roots” that grow faster and produce higher quantities of secondary metabolites than normal roots (Capone et al., 1989). The roots are kept in dark and there is no photosynthetic tissue or shoot present. This method, in addition to the industrial benefits, including reduced contamination, minimal space and maintenance requirements, and standardization, has enabled long-term laboratory cultures, effectively domesticating certain isolates.

Currently, commercial AM fungal inocula are used both in horticulture and field applications (Berruti et al., 2016). Many of the fungal propagules used in commercial products originate from TRC. The effect of TRC on the evolution of “domesticated” AM fungi is not clear (Plenchette et al., 1996). Here, we argue that commercial production of AM fungi *via* TRC represents strong selection pressure on fungi and represents a form of domestication, through changes to nutrient limitations, microbial consortia, and reduced host variation. Such selection pressure may lead to reduced genetic diversity and mutualistic quality.

## Luxurious Nutrient Conditions

Although AM fungi form functional mycorrhizas in TRC, the unique nutritional strategy of hairy roots may affect the quality of the symbiosis. In association with a normal plant, AM fungi grow in tandem with roots which fluctuate daily and seasonally (Lippu, 1998). In the case of transformed roots, the flow of carbon is consistent and continuous (Fortin et al., 2002), thereby promoting unrestricted fungal growth. In contrast, natural plants, which allocate most carbon to above ground shoots, impose carbon limits to root fungal symbionts. Root fungal symbionts of hairy roots are thus not carbon-limited. Unsurprisingly, such growing conditions promote spore production which demands significant carbon reserves (up to 60,000 per plate for *R. irregulare* DAOM197198) (Rosikiewicz et al., 2017).

In addition to luxury carbon, hairy roots experience no nutrient limitation in TRC (Bécard and Fortin, 1988). Such luxury nutrient conditions do not promote fungal provisioning of nutrients to hosts. There is considerable evidence that luxury soil nutrient status reduces both AM fungal abundance intraradically and mycorrhizal response (MR) in hosts (Menge et al., 1978; Thomson et al., 1986; Breuillin et al., 2010; Bonneau et al., 2013), which may lead to less beneficial associations. For example, increased N levels (*via* nitrogen fertilization) can select for rhizobia (Weese et al., 2015), and AM fungi (Johnson, 1993) that provide reduced benefit to the host plants. It is therefore possible that a highly eutrophic environment, such as TRC, may promote selection for less mutualistic AM fungi.

## Talk Between Microbial Neighbors

The monoxenic environment of TRC lacks much of the hyphosphere/rhizosphere microbial consortia that play an important role in the AM symbiosis. Co-existing microbes engaged in antagonistic or synergetic interactions produce bioactive compounds which can be used in defense, to confer stress tolerance or boost metabolic activities for the producers (Ola et al., 2013). Such compounds are not produced when bacteria (Koskiniemi et al., 2012) and fungi (Naiki and Cook, 1983; Gallone et al., 2016) are maintained under axenic conditions due to lack of appropriate environmental stimuli from neighboring microbes (Marmann et al., 2014). The pathways for these signaling compounds can be lost permanently *via* selective gene deletion over generations of continuous propagation *in vitro*.

Similar to other microbes, AM fungi have antagonistic and synergetic relations with other AM fungi (Wilson, 1984; Engelmoer et al., 2014) and other soil microbes (Mar Vázquez et al., 2000). For example, it was recently demonstrated that AM fungi have the ability to indirectly increase the nitrogen (N) uptake by plants *via* association with soil microbes (Hestrin et al., 2019). Therefore, growing in an environment that inhibits these interactions could reduce the effectiveness of such strains in natural conditions.

In addition to the selection pressure resulting from lack of microbial cross talk, reduction or even elimination of fungal endobacteria and bacteria that reside on the hyphal or spore surface in TRC, can affect fungal function (Dearth et al., 2018) and mutualism performance (Vannini et al., 2016). Establishing AM fungi in TRC requires surface sterilization and antibiotics in order to eliminate surface bacteria (Bécard and Fortin, 1988). However, AM fungi naturally comprise a community of bacteria that reside in, and on, hyphae and spores. Abundant rhizobia and pseudomonads have been found attached on spore and hyphal surface (Bianciotto et al., 1996b; Roesti et al., 2005; Agnolucci et al., 2015), but also bacterium-like organisms (BLOs) (Bianciotto et al., 1996a; Naumann et al., 2010) and Mollicutes/mycoplasma-related endobacteria (MRE) (Desiro et al., 2014; Torres-Cortes et al., 2015; Naito et al., 2017) were detected within the cytoplasm. Some of these bacteria possess chitinolytic abilities (Roesti et al., 2005; Agnolucci et al., 2015) and their abilities to degrade spore walls can play a crucial role in spore germination (Mayo et al., 1986). Of course, the presence of such bacteria can also benefit the colonized plants *via* a cascade of gene activation and chemical signals (Artursson et al., 2006). Long-term *in vitro* culturing could negatively affect the interaction between AM fungi and their own beneficial mutualists (Lumini et al., 2007).

## Plant Identity

There is increasing evidence that plant genotype can significantly affect the symbiosis (Chialva et al., 2018; Mateus et al., 2019). In the case of TRC propagation, fungi are exposed to dramatically reduced host diversity [most commonly carrot (*Daucus carota*) or tomato (*Solanum lycopersicum*)]. While gene activation in the early stages of colonization are preserved among hosts (Delaux et al., 2014), the progression of the symbiosis can be significantly altered depending on host identity both regarding the plant (Angelard et al., 2010) and fungal response (Cavagnaro et al., 2001; Koch et al., 2017). Mateus et al. (2019) observed large differences in the expression between fungal isolates growing on multiple cassava cultivars, but the differences were influenced largely by the genotype of the cultivar host. The reduction in host genetic diversity to a single genotype in TRC may lead to genetic drift and unused gene deletion for the AM fungus (Muller’s ratchet in host restricted lineages, see Moran et al., 2008). Recently, Sugiura et al. (2019) identified myristate, a fatty acid, as a usable carbon source from *Rhizoglomus irregulare* during the asymbiotic growth that can promote hyphal growth to the production of daughter spores in a host-free culture. While such information advances our knowledge in AM fungal metabolism, such a mechanism could also lead to host-free AM fungal propagation systems, with unknown effects on the efficacy of the symbiosis. Culturing symbionts in host-free environments has been shown to reduce symbiotic quality (Marx and Daniel, 1976; Speakman, 1982).

## Is there Evidence of Domestication on Arbuscular Mycorrhizal Fungi?

Given all the opportunities for deleterious selection on AM fungi growing in TRC, is there any evidence that domestication has happened? Evidence for domestication would require reduced genetic variation as well as morphological and functional changes.

### Reduced Genetic Variation

There is evidence that controlled conditions such as TRC can lead to loss of genetic diversity among some AM fungal isolates For example, Wyss and Bonfante (1993) showed genotypic changes among isolates of a single species (*Funneliformis mosseae* BEG12) when maintained under long-term lab conditions. In addition, there is evidence of sequence loss in spores of an isolate of a *Glomus coronatum* when maintained in cultures compared to field originated spores (Clapp et al., 2001) and reduced allelic variation in spores of *Claroideoglomus etunicatum* compared to the parent isolate following single spore inoculations (Boon et al., 2013).

### Morphological and Functional Alterations

Regardless the mechanism leading to genotype changes, there is evidence that *in vitro* cultivation affects AM fungal functional traits. *In vitro* cultivation has led to increased germination rates (Kokkoris et al., 2019b) and reduced in propagule size (Pawlowska et al., 1999; Calvet et al., 2013). Plenchette et al. (1996) found that *in vitro* produced spores of *Glomus versiforme* were significantly less infective, even only after three successive generation *in vitro*. Calvet et al. (2013) observed that *in vitro* colonization of AM fungi reduced host nutritional benefit. Similarly, Kokkoris and Hart (2019) showed that *in vitro* propagation resulted in a trade-off between spore production and phosphorus (P) benefit. Copious spore production over nutritional benefit is a trade-off that seems to be preserved even when this isolate is grown in pots with a variety of different hosts (Kokkoris et al., 2019a).

### Loss of Endobacterial Symbionts

*In vitro* cultivation may affect the endocellular bacteria associated with fungal spores. *Candidatus* Glomeribacter gigasporarum is a bacterium that resides in spores of *Gigaspora margarita*. *In vitro*, this bacterium experiences populational dilution and eventually disappears leading to “pure” spores over successive generation *in vitro* (Lumini et al., 2007). Although the bacterium is not required for *G. margarita* to complete its life cycle, its absence alters spore’s morphology and negatively affects germination and growth (Lumini et al., 2007) and can significantly alter the fungal activity (including protein expression, and quality and quantity of lipidic profile) (Salvioli et al., 2010, 2016).

### Incompatibility Between Isolates?

One potential consequence of TRC cultivation may affect hyphal fusion among compatible fungi. For example, *in vivo* cultivation for 20 years led to vegetative incompatibility for *F. mosseae* (Sbrana et al., 2018). If long-term culturing in TRC inhibits the ability of anastomosis, then domesticated isolates might be unable to interact with other isolates in nature. Incompatibility could even lead to a permanent homokaryotic stage, preventing genetic information exchange between compatible isolates and thus adaptation to novel conditions (see sidebar#2 for the importance of anastomosis on AM fungi). It could also pose a survivorship disadvantage for such an isolate if used for field inoculations due to the isolation from the natural hyphal network (Sbrana et al., 2011). Loss of anastomosis might be the reason why *in vitro* produced strains often fail to establish and persist in natural environments post inoculation (Corkidi et al., 2004; Farmer et al., 2007; Tarbell and Koske, 2007).

Sidebar#2 **The role of anastomosis in arbuscular mycorrhizal fungi.**

Heterokaryosis, first coined by Burgeff (1914), is common in the fungal kingdom. It occurs when compatible hyphae fuse (anastomosis), but do not undergo karyogamy. Heterokaryosis is observed in earlier-diverging lineages of fungi such as Phycomyces (Mucoromycota), where most sexual spores contain three to four nuclei, which can be genetically similar or different (Mehta and Cerdá-Olmedo, 2001). The classical understanding of heterokaryosis emphasizes the dikaryon as a precursor to karyogamy, or the eventual fusing of two nuclei to form a monokaryotic, diploid cell, which then undergoes meiosis leading to the production of haploid, sexual spores. However, there are many examples of fungi for which the heterokaryon plays a role beyond sexual recombination [Jinks, 1952 (*Penicillium*); James et al., 2008 (*Heterobasidion*); Roper et al., 2011; Samils et al., 2014 (*Neurospora*)] Diverse nucleotypes in spores may prevent the loss of genetic variation and functional diversity in the case of population reduction (i.e., prevent genetic drift through bottleneck effects) or ensure maintenance of genetic information when new populations are established from few propagules (prevent genetic drift through founder effects).

Similar to other heterothallic fungal groups (Asco- and Basidiomycotan), nucleotype diversity may be maintained through anastomosis in wild populations of closely related AMF (Giovannetti et al., 1999, 2001, 2003; Croll et al., 2008). For AM fungi, anastomosis has been observed among hyphae of the same isolates during the asymbiotic and symbiotic stage even when growing in different systems but also been observed between closely related isolates (Giovannetti et al., 2001; Croll et al., 2009; Purin and Morton, 2011; De la Providencia et al., 2013; de Novais et al., 2013; Barreto De Novais et al., 2017). During anastomosis, plasmogamy can occur, and mitochondria (De la Providencia et al., 2013) and nuclei (Croll et al., 2008) can be shared between partners. Although anastomosis is recognized primarily as a healing mechanism post disturbance (de la Providencia et al., 2005) especially for *Gigaspora* sp., also plays an important ecological role, since newly germinating spores can connect to the pre-established network prior interacting with a host, gaining an important survivorship benefit (Sbrana et al., 2011).

While debate about their status as homo- versus heterokaryons has circulated for nearly two decades (Kuhn et al., 2001; Hijri and Sanders, 2004; Pawlowska and Taylor, 2004; Croll et al., 2008; Tisserant et al., 2013; Lin et al., 2014; Boon et al., 2015), recent evidence shows that AM fungi can be haploid and homokaryotic (meaning that they contain thousands of genetically similar nuclei with haploid number of chromosomes) or haploid and dikaryotic (meaning that they contain two genetically distinct type of nuclei with in equal proportions, with a haploid number of chromosomes) (Ropars et al., 2016; Chen et al., 2018a). Corradi and Brachmann (2017) proposed that compatible AM fungi have the ability to mix their nuclei continually in the field, through anastomosis, effectively creating genetically novel isolates *via* karyogamy and meiosis. Although it is still not yet clear under which conditions the dikaryons proceed to karyogamy and meiosis aka to sexual reproduction (Chen et al., 2018b), hyphal fusion between compatible isolates seem to represent a vital step for plasmogamy and exchange of genetic information.

## Can We Overcome Transformed Root Cultures-Induced Domestication?

If TRC propagation of AM fungi produces inferior mutualists, it is reasonable to wonder whether specific practices in TRC production could prevent such unwanted changes. Such practices, like co-cultivation, medium modifications and re-association with natural hosts, already exist and applied in other microbial systems.

## Co-Cultivation with Microbes

Axenic, and in case of AM fungi, monoxenic growing conditions can reduce the chemical diversity of the produced compounds due to lack of environmental stimuli. Co-cultivation of microbes can activate silent gene clusters of the microbial partners (Brakhage et al., 2008), protecting fungi against genetic drift and gene deletion. For example co-culture of bacteria and fungi (*Fusarium tricinctum Bacillus subtilis*) showed a 78-fold increase in fungal metabolite production compared to the pure culture of the fungus (Ola et al., 2013). Similarly, a fungal co-culture (*Coprinopsis cinerea* and *Gongronella* sp.) produced 900 times increased oxidation activity compared to pure cultures (Pan et al., 2014). Growing AM fungi with two *Paenibacillus validus* bacterial isolates increased fungal growth even in absence of a host (Hildebrandt et al., 2002). Co-cultivation of AM fungi with diverse microorganisms may be a way to maintain genetic variation and function by activating AM fungal genes that would otherwise be silent, due to reduced environmental stimuli, and prone to deletion if maintained long term in TRC.

## Culturing Conditions

The most commonly used TRC medium is the M medium proposed by Bécard and Fortin (1988). While no major modifications have been made on M medium, addition of simulative chemical molecules could compensate for lack of microbial associates and trigger gene activation for secondary metabolite production. For example, the addition of fatty acids (signal from *P. validus*, see previous section) can induce colonization ability and stimulate the spore production of AM fungi (Kameoka et al., 2019). In addition, chemical effectors responsible for promoting hyphal branching, mycorrhization, and the efficiency of the symbiosis have been identified (e.g., strigolactones) (Akiyama et al., 2005; Besserer et al., 2006), which may lead to increased gene activation and help maintaining the genetic and functional variation in TRC.

Additional changes in the medium or in the growing conditions may stimulate recombination in AM fungi and encourage the production of novel genotypes. For example, in *Coprinus congregatus*, a Basidiomycete, the quantity of arginine in the medium controls the expression of the mating type genes and ultimately the growth of the fungus as a homo or dikaryon (Ross et al., 1991). We need to identify environmental controls of AM fungal mating behavior to optimize growing conditions that will choose for efficient symbionts and not just copious spore producers.

## Re-Association with Compatible Hosts

Ectomycorrhizal (ECM) fungi can lose their symbiotic ability and eventually fail to colonize plant roots if maintained *in vitro* long term (Marx and Daniel, 1976). Growing strains *via* host passage (association with a compatible host) every 4 years alleviates this bottleneck (Marx, 1981). The re-isolated strain from the colonized roots shows increased colonization ability but also increased symbiotic quality compared to solely *in vitro* retained strains (Thomson et al., 1993). Similarly for pathogenic fungi, pathogenicity can be lost with long-term *in vitro* cultivation and “passaging” the strains trough a compatible host and re-isolating and can revitalize their infective abilities (Speakman, 1982). Furthermore, the “asexual” yeast *Candida albicans* was stimulated to mate when injected into a mammalian host (Hull et al., 1999) showing the significant role an appropriate host can have even for sexual reproduction. While the presence of a root system is a prerequisite for AM fungal cultures, the important differences between TRC and real plant system may alter the AM fungal function. Passage through real host or even community of hosts could retain the functionality of the domesticated strains.

## Conclusions

There is clear evidence that continuous *in vitro* propagation alters AM fungal morphology, genetics, and functioning, meaning that domestication of such strains is in progress or has occurred. While mass production of AM fungal propagules is needed for a sustainable inoculant industry, *in vitro* propagation may bring unwanted changes to the cultured isolates. If domestication reduces the isolate’s ability to anastomose, these fungi would have a fitness disadvantage in the field. Alternatively, if the unnatural environment of TRC creates strains that are less beneficial in natural conditions, but these isolates are still able to anastomose with native fungi, such isolates may impact negatively on the gene pool of natural populations. It is important to further examine the effects of domestication on AM fungi and predict how changes could greatly affect the environment following inoculation with such strains.

## Data Availability Statement

No datasets were generated or analyzed for this study.

## Author Contributions

MH and VK conceptualized the work and shared the writing and revision of the MS. MH and VK approved the publication of the MS in its current form. MH and VK agreed to be accountable for all aspects of the work including accuracy or integrity of any part of the work.

### Conflict of Interest

The authors declare that the research was conducted in the absence of any commercial or financial relationships that could be construed as a potential conflict of interest.

## Abbreviations

AM: Arbuscular mycorrhizas/arbuscular mycorrhizal
BLOs: Bacterial like organisms
ECM: Ectomycorrhizas/ectomycorrhizal
MRE: Mollicutes/mycoplasma-related endobacteria
MR: Mycorrhizal response
N: Nitrogen
P: Phosphorus
Ri: Root inducing
TRC: Transformed root cultures.
